# Zinc-mediated structural and functional regulation of ERp44 and ERGIC-53 in protein quality control

**DOI:** 10.1093/jb/mvag033

**Published:** 2026-05-05

**Authors:** Satoshi Watanabe, Kenji Inaba

**Affiliations:** Medical Institute of Bioregulation, Kyushu University, Maidashi 3-1-1, Higashi-ku, Fukuoka 812-8582, Japan; Medical Institute of Bioregulation, Kyushu University, Maidashi 3-1-1, Higashi-ku, Fukuoka 812-8582, Japan

**Keywords:** cargo transport, ERGIC-53, ERp44; MCFD2, protein quality control, zinc

## Abstract

Zinc ions (Zn^2+^) are essential trace metal ions in the human body. Intracellular Zn^2+^ levels are tightly regulated by two metal transporter families: ZIPs, which mediate Zn^2+^ influx into the cytosol, and ZnTs, which export Zn^2+^ from the cytosol to the extracellular space or sequester it into the cellular organelles. Within cells, Zn^2+^ plays multiple roles, acting as a catalytic cofactor for numerous enzymes, stabilizing protein structures, and functioning as a second messenger in signal transduction. In addition, Zn^2+^ is involved in the transient regulation of enzymatic activities. Here, we review recent findings that reveal novel roles of Zn^2+^ in the structural and functional regulations of the molecular chaperone ERp44 and the cargo receptor ERGIC-53, both of which operate for protein quality control in the early secretory pathway.

## Graphical Abstract

**Graphical Abstract:**
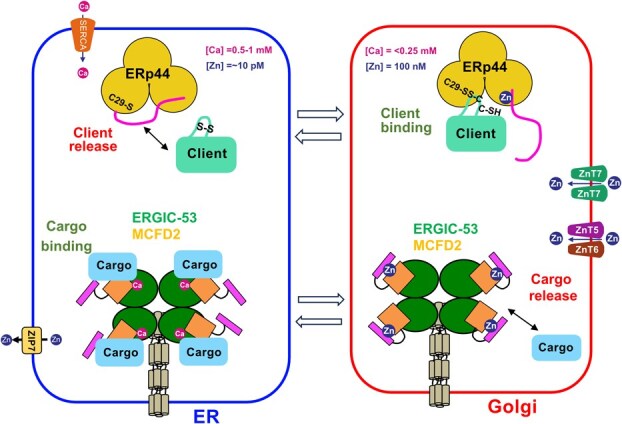

Zinc is essential and the second most abundant trace metal in cells, after iron *(**1**,*  *2**)*. Zinc ions (Zn^2+^) play a crucial role by acting as essential cofactors of various enzymes, including metalloproteases, and by serving as structural elements that stabilize the three-dimensional structures of proteins, such as zinc-finger DNA-binding proteins and RING-finger ubiquitin ligases *(**3**)*. Furthermore, free labile Zn^2+^ serves as a second messenger in signal transduction *(**4**,*  *5**)*. Intracellular Zn^2+^ levels are maintained by two families of zinc transporters: ZnT (zinc transporter, SLC30) and ZIP (Zrt/Irt-like protein, SLC39) *(**6–9**)*. ZIP family members transport Zn^2+^ from the extracellular space or organelles into the cytosol, whereas ZnT family transporters mediate Zn^2+^ transport in the opposite direction. Four ZnT family members (ZnT4, 5/6 and 7) and three ZIP family members (ZIP9, 11 and 13) are localized in the Golgi apparatus, suggesting that the dynamic import and export of Zn^2+^ occur around this organelle *(**10**,*  *11**)*. Zn^2+^ is incorporated into secretory metalloenzymes during the protein transport along the secretory pathway *(**12**,*  *13**)*. In this review, we provide an overview of recent structural and mechanistic insights into emerging physiological roles of Zn^2+^ in the regulation of molecular chaperones and cargo receptors that cycle between the Endoplasmic Reticulum (ER) and Golgi, processes essential for proteostasis in the early secretory pathway (ESP).

## ERp44 for the Second Checkpoint of Protein Quality Control

In the ER, chaperones such as BIP, members of the protein disulfide isomerase (PDI) family, and glycosyltransferases catalyse the folding, glycosylation and maturation of nascent secretory proteins *(**14–16**)*. To maintain proteostasis in the ESP, BiP and calnexin/calreticulin serve as primary quality-control checkpoints that only allow properly folded proteins to proceed to the Golgi *(**17**)*. Importantly, ERp44, a member of the PDI family, is mostly localized in the ERGIC and *cis*-Golgi and functions as the second checkpoint that surveys proteins transported to the Golgi *(**18**,*  *19**)*. This chaperone, in collaboration with KDEL receptors, facilitates the retrograde transport of immature oligomeric secretory proteins, such as immunoglobulin M (IgM) *(**20**)* and adiponectin *(**21**)*, from the Golgi to the ER. Its targets also include several ER-resident enzymes that do not have ER-retrieval motifs like a C-terminal KDEL sequence. This group includes ER oxidoreductin-1 (Ero1) *(**22–24**)*, ER aminopeptidase 1 (ERAP1) *(**25**)* and peroxiredoxin 4 (Prx4) *(**26**,*  *27**)*.

**Fig. 1:**
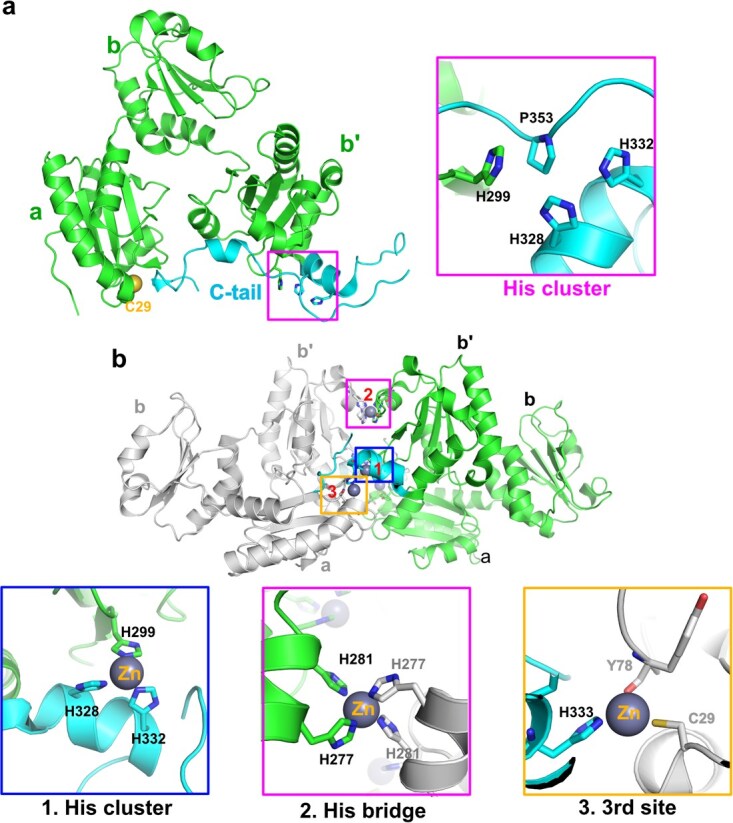
**Structures of ERp44 in Zn**  ^**2+**^**-free and Zn**  ^**2+**^**-bound states.** (A) Crystal structure of ERp44 in the metal-free state (PDB ID: 5GU6). The inset shows a close-up view of the histidine cluster (His cluster). (B) Crystal structure of an Zn^2+^-bridged homodimer of ERp44 (PDB ID: 5XWM). The insets show close-up views of three Zn^2+^ binding sites in ERp44.

The overall structure of ERp44 consists of three thioredoxin (Trx)-like domains (**a**, **b** and **b′**), followed by a C-terminal extension named C-tail (Fig. 1A) *(**28**,*  *29**)*. The **a** domain contains a unique CRFS motif, in which Cys29 forms a mixed disulfide bond with client proteins *(**22**)*. Previous studies have shown that ERp44 interacts with its target clients in a pH-dependent manner *(**30**)*. At the neutral pH of the ER, the C-tail of ERp44 adopts a closed conformation, shielding the client-binding site around Cys29 through multiple hydrogen bonds with residues in the **a** domain *(**29**)*. In contrast, within the weakly acidic Golgi, protonation of conserved histidine residues of ERp44 triggers domain rearrangement and partial opening of the C-tail, thereby promoting efficient formation of mixed disulfide bonds with client proteins *(**29**)*.

## Histidine for Zn^2+^ Binding in ERp44

A high-resolution structure of ERp44 revealed that His299, His328 and His332, which are highly conserved among ERp44 orthologues, are spatially clustered and together form a histidine cluster (His cluster) *(**29**)* (Fig. 1A inset). Given that histidine residues frequently participate in the coordination of heavy metal ions including Zn^2+^, Fe^2+^ and Mn^2+^, the presence of this His cluster suggests that ERp44 has the capacity to bind these metal ions. Our extensive ITC analysis revealed that ERp44 specifically binds Zn^2+^ with apparent *K*_d_ values of 135 to 295 nM at pH 7.2 to 6.2 *(**31**)*.

The crystal structure of Zn^2+^-bound ERp44 revealed that ERp44 forms an Zn^2+^-bridged homodimer, in which Zn^2+^ is coordinated by two histidine pairs (His277 and His281) of the **b′**domains from two ERp44 molecules at the centre of the homodimer (Fig. 1B, middle lower) *(**31**)*. As expected, Zn^2+^ is also bound to the His cluster (Fig. 1B, left lower). In addition, the Zn^2+^-bridged homodimer is stabilized by a third Zn^2+^ binding site formed by His333 of one protomer, together with Cys29 and the main-chain carbonyl oxygen of Tyr74 of the other protomer (Fig. 1B, right lower). This third Zn^2+^-binding site appears to play an auxiliary role in stabilizing the ERp44 homodimer.

## Zn^2+^ Binding to the His Cluster Promotes the Complex Formation Between ERp44 and Clients

**Fig 2:**
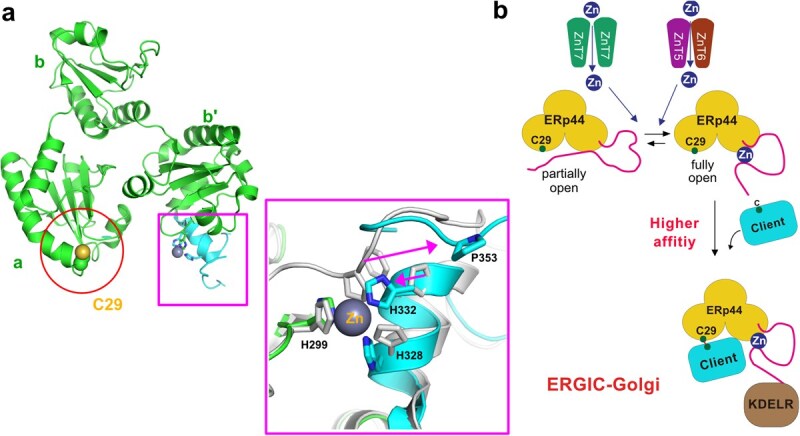
**Zn**  ^**2+**^**-dependent activation of ERp44.** (A) Open conformation of Zn^2+^-bound ERp44, in which the client-binding surface including Cys29 (red circle) is exposed to the solvent. The inset highlights a close-up view of the local conformational changes in the His cluster upon Zn^2+^ binding. (B) Schematic illustration of Zn^2+^-dependent activation of ERp44, mediated by Golgi-resident Zn^2+^ transporters, ZnT7 and ZnT5/6.

In the metal-free state, the His cluster is interrupted by Pro353 and thereby adopts a non-optimal configuration for Zn^2+^ binding (Fig. 1A inset). Upon Zn^2+^ binding, Pro353 is largely displaced, and His299 moves closer to His328 and His332, leading to tight coordination of Zn^2+^ at the His cluster. The displacement of Pro353 triggers a large movement of the C-tail toward the **b′**domain, leading to C-tail opening (Fig. 2A). Consequently, the client-binding site, including Cys29, becomes fully exposed to the solvent. Accordingly, Zn^2+^-bound ERp44 exhibits a higher affinity for its target clients than the metal-free form (Fig. 2B). Thus, Zn^2+^ binding to the His cluster enables ERp44 to capture its client proteins with enhanced affinity.

## Zn^2+^-Dependent Cellular Function of ERp44 via ZnT Transporters

The Zn^2+^-binding ability of ERp44 suggests that its cellular functions are regulated in an Zn^2+^-dependent manner *(**31**)*. The ER is characterized by neutral pH and picomolar levels of free Zn^2+^  *(**32**)*. Under these chemical conditions, ERp44 predominantly adopts a closed conformation with the C-tail masking its client-binding surface (Fig. 1A). In contrast, the labile Zn^2+^ concentration in the Golgi is maintained at approximately 100 nM *(**10**)*, under which ERp44 binds Zn^2+^ and adopts a fully open conformation that exhibits a higher affinity for its target clients (Fig. 2A and B).

In the secretory pathway, four ZnT family members are localized to import Zn^2+^ into the Golgi lumen. Among them, ZnT7 localizes to the pre*-cis* and *cis*-Golgi and plays a primary role in the proper localization of ERp44 by supplying Zn^2+^ to ERp44 *(**10**)*. The ZnT5/6 heterodimer and the ZnT4 homodimer mainly localize downstream of ZnT7 and are also involved in the regulation of ERp44. Single knockdown of ZnT7, as well as simultaneous knockdown of these ZnT transporters caused marked Golgi accumulation of ERp44 and reduced ERp44 activity *(**10**)*. Thus, the concerted actions of the ZnT family members ensure Zn^2+^-dependent protein quality control mediated by ERp44 (Fig. 2B).

**Fig. 3:**
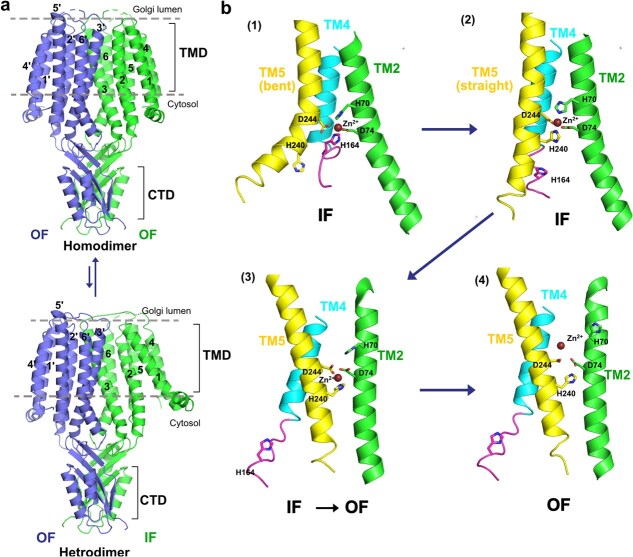
**Structures and Zn**  ^**2+**^**-transport mechanism of human ZnT7.** Cryo-EM structures of an ZnT7 homodimer, in which both protomers adopt an OF conformations (upper), and an ZnT7 heterodimer featuring one inward-facing (IF) and one OF protomer (lower). Molecular mechanism of ZnT7-mediated Zn^2+^ transport.

Importantly, we determined cryo-EM structures of human ZnT7 in Zn^2+^-bound and Zn^2+^-unbound forms *(**33**)*. Overall structure of ZnT7 adopts a ‘mushroom’-shaped dimeric architecture (Fig. 3A). Each protomer of ZnT7 consists of N-terminal six transmembrane helices (TM1–TM6) and C-terminal cytosolic domain (CTD) comprising two α helices and four β-strands, with the latter contributing to the dimer formation. In the Zn^2+^-unbound form, human ZnT7 forms a homodimer in which both protomers adopt an outward-facing (OF) conformation as a major state, as well as an asymmetric heterodimer composed of one inward-facing and one OF protomer as a minor state (Fig. 3A, lower). Notably, the Zn^2+^-bound structures determined under various conditions reveal that ZnT7 transports Zn^2+^ through a step-by-step mechanism (Fig. 3B). The IF protomer in which TM5 adopts an open and bent conformation initially captures Zn^2+^ through three residues of the conserved HDHD motif (His70, Asp74 and Asp244) together with a histidine residue (His164) in the long histidine-rich loop (Fig. 3B, step 1). Subsequently, His164 is displaced from the bound Zn^2+^, and His240 of the HDHD motif instead coordinates the Zn^2+^ through straightening of TM5 (Fig. 3B, step 2). The protomer then undergoes an IF to OF conformational transition (Fig. 3B, step 3). During this transition, His70 gradually move away from the bound Zn^2+^. Consequently, Zn^2+^ is released into the Golgi lumen, likely facilitated by protonation of His70 and His240 (Fig. 3B, step 4).

## ERGIC-53, a Receptor for Cargo Transport From the ER to the Golgi

In the ESP, subsets of newly synthesized secretory and membrane proteins are efficiently transported from the ER to the Golgi with the aid of cargo receptors *(**34**)*. ERGIC-53 (also named LMAN1) and SURF4 function as major cargo receptors mediating protein transport between the ER and the Golgi *(**35**,*  *36**)*. Target cargo proteins of ERGIC-53 include coagulation factor V (FV) and factor VIII (FVIII) *(**37**,*  *38**)*, α1-antitrypsin *(**39**)*, several membrane proteins like GABA_A_ receptor and NDST1 *(**40**,*  *41**)*, and surface glycoproteins of infected RNA viruses *(**42**)*_._ Mutations in the *ergic-53* gene and its functional partner, the *multiple coagulation factor deficiency* 2 gene (MCFD2) *(**43**,*  *44**)*, cause a genetic bleeding disorder known as combined FV and FVIII deficiency (F5F8D).

ERGIC-53 comprises a luminal carbohydrate recognition domain (CRD), a long stalk domain, a transmembrane (TM) helix and a short cytoplasmic tail (CT) *(**45**)*. The CRD exhibits both structural and functional similarities to plant L-type lectins and recognizes high-mannose type glycans on target cargo proteins in a Ca^2+^-dependent manner *(**46–49**)*. The KKFF motif at the C-terminus is essential for COPII-mediated ER exit (FF motif) and COPI-dependent ER retrieval (via the KK motif), thereby allowing ERGIC-53 to cycle between the ER and the Golgi *(**50**,*  *51**)*. The stalk domain comprises four long α-helices, which are predicted to form a coiled coil. On the other hand, MCFD2 is a compact protein characterized by an EF-hand domain that contains two calcium-binding sites and functions as a cargo adaptor for ERGIC-53, assisting Ca^2+^-dependent capture of cargo proteins in the ER *(**52**)*. Most missense mutations causing F5F8D are located in the EF-hand domain of MCFD2 and destabilize its three-dimensional structure *(**37**)*.

## Tetrameric Structure of Full-Length ERGIC-53 in Complex with MCFD2

**Fig. 4:**
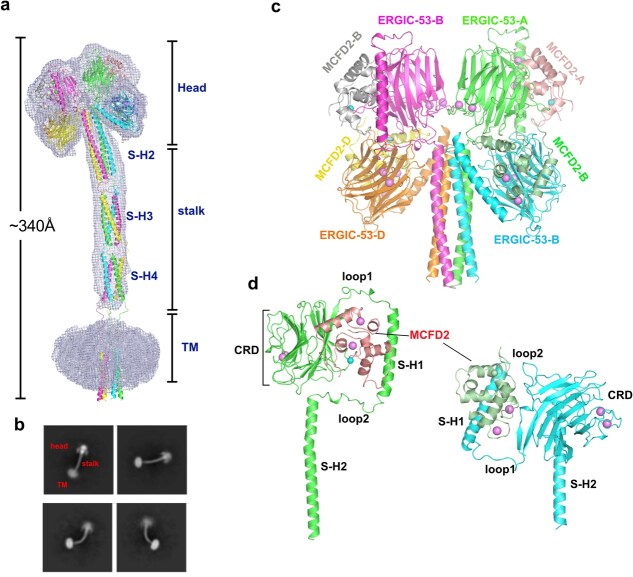
**Structure of ERGIC-53 complexed with MCFD2.** (A) Cryo-EM map and model of full-length ERGIC-53 in complex with MCFD2 (PDB ID:8JPG). (B) Representative 2D class-averaged images of full-length ERGIC-53 showing straight and bent conformations. (C) Cryo-EM structure of the tetrameric head region of ERGIC-53. Magenta and cyan spheres represent bound calcium and zinc ions, respectively. (D) Two different configurations of ERGIC-53 protomers are shown: an upper configuration, in which the CRD is positioned above the S-H2 (left), and a lower configuration in which the CRD is positioned below the top of the S-H2 (right). MCFD2 is bound to the cleft formed by the CRD, S-H1, and two loops.

Recent cryo-EM structures of full-length ERGIC-53 complexed with MCFD2 revealed that ERGIC-53 exists as a homotetramer, not a homohexamer as previously suggested, adopting a four-leaf clover-like architecture (Fig. 4A) *(**53**)*. The overall structure of ERGIC-53 consists of a tetrameric head, a long stalk domain composed of three sets of four-helix coiled-coils, and a single-pass TM domain (TMD) with the CT. 2D class-averaged images showed that in some particles, the stalk domain assumed nearly straight conformations, whereas in others, it exhibited largely bent conformations at various angles, suggesting a high degree of flexibility in the stalk region of ERGIC-53 (Fig. 4B).

High-resolution cryo-EM analysis focusing on the head region revealed its detailed conformational architecture. The head region is composed of the CRD, stalk helices 1 and 2 (S-H1 and S-H2) (Fig. 4C and D). S-H2 forms a long four-helix coiled coil at the centre of the tetramer. Two of the four protomers adopt an upward configuration, wherein the CRD and S-H1 are positioned above S-H2 (Fig. 4D). In contrast, the other two protomers assume a downward configuration, where CRD and S-H1 are rotated by approximately 180° around the SH1–SH2 loop, positioning them beneath the top of S-H2 (Fig. 4D). The upper and lower CRDs associate vertically to form a two-layered CRD dimer, which further dimerizes to generate the four-leaf clover-like tetramer.

## Zn^2+^ Binding Site of MCFD2 Involved in Regulation of FV Binding

**Fig. 5:**
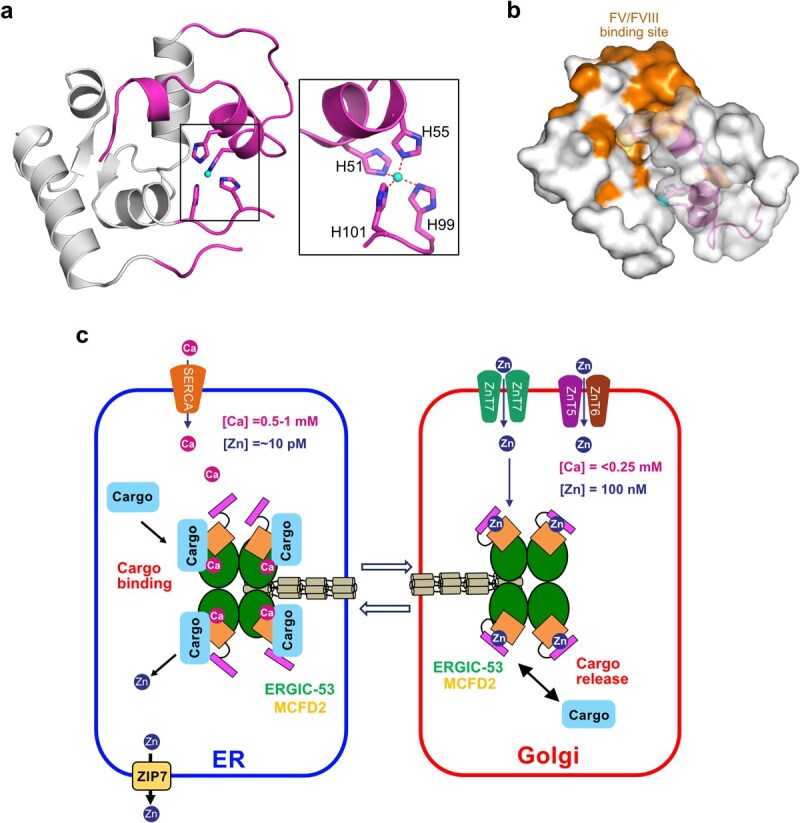
**Zn**  ^**2+**^**-dependent regulation of MCFD2. (**A) Cryo-EM structure of MCFD2 within the complex with ERGIC-53. The region missing in the previous crystal structures is shown in magenta. The inset highlights Zn^2+^ coordination by four histidine residues of MCFD2. (B) Surface representation of MCFD2 with the FV/VIII binding site coloured in orange, highlighting partial masking of the cargo-binding site by the Zn^2+^-stabilized N-terminal lid of MCFD2. (C) Schematic illustration of Ca^2+^- and Zn^2+^-dependent regulation of cargo binding and release by the ERGIC-53–MCFD2 complex (see text for details).

In the cryo-EM structure of the ERGIC-53–MCFD2 complex, MCFD2 binds to a cleft formed by the CRD, S-H1 and two long loops (loop 1 and loop 2), forming a 1:1 complex with an ERGIC-53 protomer (Fig. 4C and D). Several missense or deletion mutations that disrupt the interaction between ERGIC-53 and MCFD2 are also known to cause F5F8D *(**37**,*  *54**)*. The N-terminal segment of MCFD2, which was disordered in the original crystal structures of its complex with the CRD of ERGIC-53 *(**55**,*  *56**)*, contains two short α-helices that associate with the EF-hand domain (Fig. 5A). Of note, four conserved histidine residues (His51, His55, His99 and His101) are located in close proximity to form a His cluster that coordinates an Zn^2+^ ion (Fig. 5A, inset).

MCFD2 has been reported to recognize specific sequences within the B domains of FV and FVIII, termed passport motifs (PS), which regulate ER-to-Golgi transport and promote N-glycan maturation of PS-containing glycoproteins *(**57**,*  *58**)*. In the Zn^2+^-bound state of MCFD2, the surface involved in binding PS is partially masked by the N-terminal short a-helices stabilized by the bound Zn^2+^ (Fig. 5B). These structural features suggest that Zn^2+^ regulates the opening and closing of the N-terminal segments of MCFD2, which act as a lid controlling FV/FVIII binding and release. Consistent with this, the addition of exogenous Zn^2+^ to the culture medium significantly interfered with the secretion of FV *(**53**)*. Thus, Zn^2+^ binding to MCFD2 restricts the association of cargo proteins with MCFD2 and ERGIC-53.

## Ca^2+^- and Zn^2+^-Dependent Regulation of Cargo Binding and Release

The ability of MCFD2 to bind both Ca^2+^ and Zn^2+^ raises the possibility that cargo binding and release by the ERGIC-53–MCFD2 complex are regulated by these two metal ions (Fig. 5C). Whereas Ca^2+^ is present in the ER at millimolar concentrations (0.5–1 mM) *(**59**)*, labile Zn^2+^ is tightly regulated and maintained at picomolar levels in this organelle *(**32**)*. Under these conditions, Ca^2+^ binds to the EF hand domain of MCFD2, contributing to its proper folding. Meanwhile, Zn^2+^ does not bind to the His cluster, leaving the N-terminal lid in an open conformation that fully exposes the cargo-binding site. Ca^2+^ also binds to the CRD of ERGIC-53, thereby stabilizing its glycan binding site *(**56**,*  *60**)*. Consequently, the ERGIC-53 complexed with MCFD2 can efficiently capture its target cargo proteins.

Once formed, the ternary complex composed of ERGIC-53, MCFD2 and a cargo protein is transported to the Golgi via the COPII machinery. In the Golgi, Zn^2+^ transporters, ZnT7 and ZnT5/6, actively import Zn^2+^ into the lumens of the pre-*cis-, cis-,* and medial-Golgi cisternae, contributing to maintaining labile Zn^2+^ concentrations at 50–100 nM *(**10**)*. Under these conditions, Zn^2+^ likely binds to the His cluster of MCFD2, resulting in the closure of the N-terminal lid region. Simultaneously, Ca^2+^ concentrations are decreased in the Golgi, compared to those in the ER, destabilizing the glycan-binding site within the ERGIC-53–MCFD2 complex *(**47**)*. As a result, the cargo protein is readily released from ERGIC-53 and thereafter transported to downstream compartments. Thus, the ERGIC-53–MCFD2 complex utilizes the pronounced difference in both Ca^2+^ and Zn^2+^ concentrations between the ER and the Golgi to regulate cargo binding and release.

## Perspectives on Zn^2+^-Mediated Regulation of Biomolecules

In the ESP, Zn^2+^ has also been reported to modulate other chaperones, including PDI dimerization *(**61**)* and the interaction between ERp57 and calnexin *(**62**)*, and to participate in the regulation of the ER redox environment *(**63**)*, suggesting that maintaining proper Zn^2+^ concentrations is critical for proteostasis. In this context, the expression of ZnT5, ZnT6 and ZnT7 is upregulated in colorectal and gastric cancers *(**64**,*  *65**)*. Thus, manipulation of Zn^2+^ transporters and Zn^2+^-dependent chaperone activity represents a potential therapeutic strategy for cancer.

In addition to the ERp44 and ERGIC-53–MCFD2 systems, several enzymes are known to be allosterically regulated by Zn^2+^  *(**66**)*. In Caspase-6, cysteine–aspartic proteases involved in apoptosis, Zn^2+^ is bound to an exosite composed of glutamate, histidine and lysine residues, inducing conformational changes that impair the catalytic site *(**67**)*. Similarly, Zn^2+^ binding to the Gla domain of protein C induces conformational changes, leading to enhanced association with the endothelial cell protein C receptor *(**68**)*.

In general, these allosteric Zn^2+^ binding sites, including those in ERp44 and MCFD2, exhibit a moderate affinity for Zn^2+^ compared to catalytic and structural Zn^2+^ binding sites *(**66**)*, allowing this metal ion to act as a transient modulator. Further structural and biochemical studies of His cluster-like motifs, in combination with AlphaFold-based structure prediction *(**69**)* or *de novo* computational design approaches will expand our understanding of Zn^2+^-dependent regulation and its potential biological and biomedical applications.
